# Assessment of Vitamin D Levels and Its Correlation With Osteoporosis and Fracture Site Comminution in Osteoporotic Hip Fractures in Tertiary Care Hospital

**DOI:** 10.7759/cureus.12982

**Published:** 2021-01-29

**Authors:** Kishore Vellingiri, Prabhu Ethiraj, Nagakumar J S., Arun H Shanthappa, Karthik S J.

**Affiliations:** 1 Department of Orthopaedics, Sri Devaraj Urs Medical College, Sri Devaraj Urs Academy of Higher Education and Research, Kolar, IND

**Keywords:** vitamin-d deficiency, osteoporotic hip fractures

## Abstract

Introduction: A global concern is vitamin D deficiency and insufficiency. There is a particularly high risk for pregnant women, people of color (Blacks, Hispanics, and those with increased skin melanin pigmentation), obese children and adults, and children and adults who are abstinent from direct sun exposure. The goal of this study was to understand the incidence of vitamin D deficiency in patients with osteoporotic hip fractures in our rural population and also to know its association with osteoporosis and osteoporotic hip fractures in a tertiary care trauma center.

Methods: This prospective research was performed at our tertiary trauma treatment center in Kolar, Karnataka, India by the Department of Orthopedics from September 2019 and July 2020. The age category was 45-90 years, intertrochanteric fractures were graded using the Boyd and Griffin classification and femoral neck fractures the Garden's staging. The research included all patients with fractures after a trivial trauma such as slip and fall while standing/walking and excluded patients with a serious history of trauma such as road traffic accidents/falls from height and pathological fractures. This study involved 30 patients. Age and gender, type of fracture, vitamin D levels, Singh’s index, and comminution of fracture site were reported in patient demographics.

Results: Thirty patients comprised the study population. Most of them were female. Females comprised 18 out of the 30. There were twelve, thirteen, and five persons in our sample population between the ages of 45-60, 61-75, and >75 years. The Singh's index was tabulated. Mean vitamin D levels were 9.64+/-3.23 in the femur fracture group and 13.42+/-5.31 in the intertrochanteric fracture group. Mean levels of vitamin D are included as a graphical representation. The comminution of the fracture site in groups of the femur and intertrochanteric fractures was eight and six, respectively.

Conclusion: Early diagnosis and treatment of these patients with vitamin D for osteomalacia and anti-osteoporotic osteoporosis regimens will hopefully enhance bone, muscle, and general health, minimizing falls and fractures.

## Introduction

Global concern is vitamin D deficiency and insufficiency [1]. There is a particularly high risk for pregnant women, people of color (Blacks, Hispanics, and those with increased skin melanin pigmentation), obese children and adults, and children and adults who are abstinent from direct sun exposure [2]. Using the Chemiluminescence Immuno Assay, serum levels of 25-hydroxyvitamin D (25-OH vitamin D) were analyzed in the laboratory using an automated analyzer. Vitamin D less than 20 ng/ml serum level was considered to be deficient. Vitamin D level was considered to be inadequate between 20 and 29 ng/ml called vitamin D deficiency (hypovitaminosis D) and Vitamin D levels were considered normal between 30 and 100 ng/ml [3]. Evidence-based screening methods can increase the detection of patients who are most likely to benefit from fracture prevention drug treatment. Furthermore, thorough consideration of when to start pharmacotherapy and the option of medication and treatment period can optimize the benefits of fracture prevention while minimizing the possible harms of long-term drug exposure [4]. The goal of this study was to understand the incidence of vitamin D deficiency in patients with osteoporotic hip fracture in our rural population and also to know its association with osteoporosis and osteoporotic hip fractures in a tertiary care trauma center.

This research was accepted at the 8th International Conference on Health and Medicine, 30 January 2021, in Colombo, Sri Lanka, for a virtual presentation. The abstract of this article will be published in the conference online supplement.

## Materials and methods

This prospective research was performed at our tertiary trauma treatment center in Kolar, Karnataka, India by the Department of Orthopedics from September 2019 and July 2020. The institutional ethics committee approved this study. The age category was 45-90 years. Intertrochanteric fractures were graded using the Boyd and Griffin classification and femoral neck fractures the Garden's staging. The research included all patients with fractures after a trivial trauma such as slip and fall while standing/walking and excluded patients with a serious history of trauma such as road traffic accidents/falls from height and pathological fractures. This study involved 30 patients. Age and gender, type of fracture, vitamin D levels, Singh’s index, and comminution of fracture site were reported in patient demographics. The information collected was coded and entered into an Excel file. The mean and standard deviation were calculated. To determine the statistical significance, the t-test was used for continuous variables and the Chi-square test for categorical variables. A p-value <0.05 was considered statistically significant.

## Results

**Figure 1:**
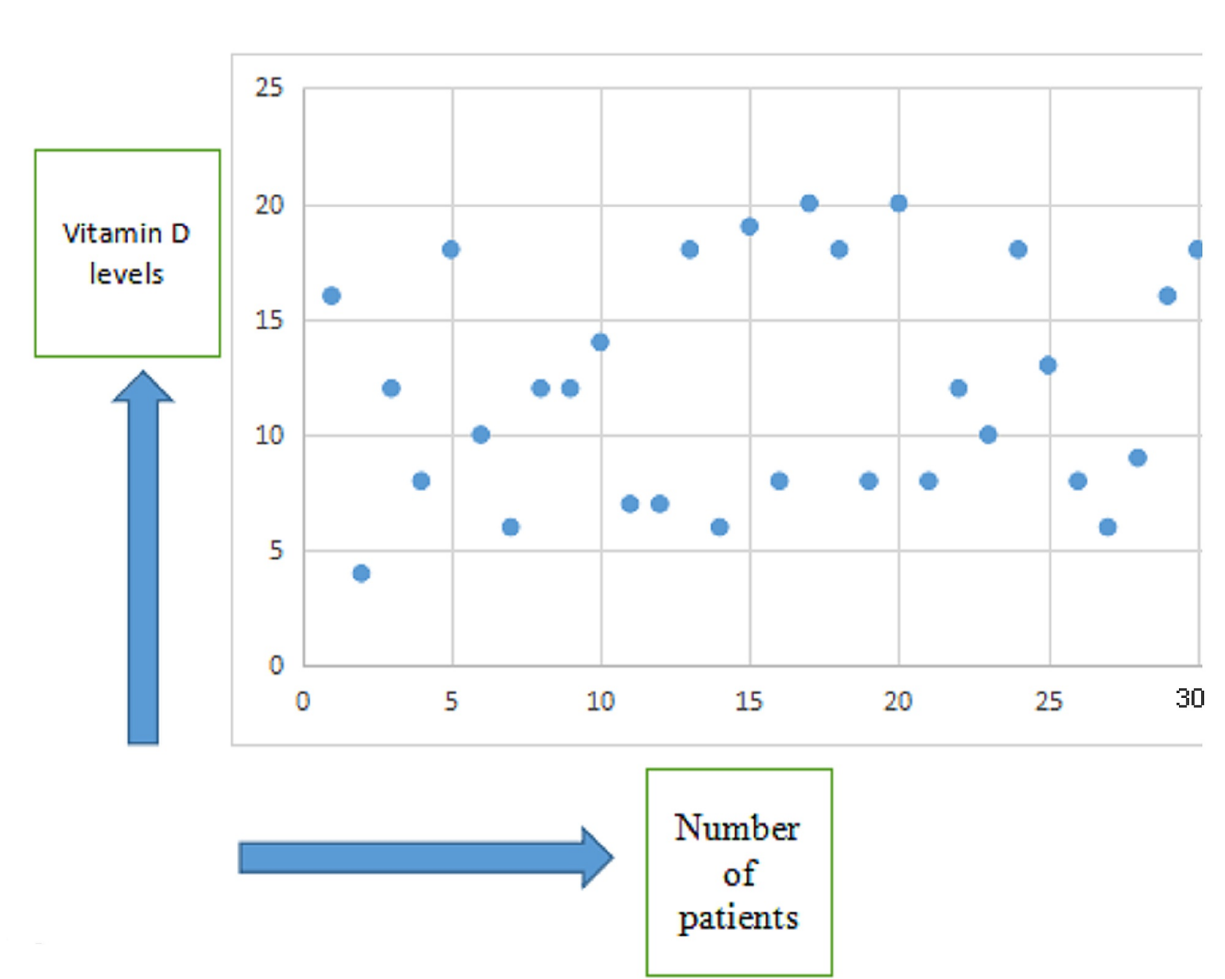
Scatter plot showing vitamin D levels in our study population

Thirty patients comprised the study population. Most of them were females. Females comprised 18 out of the 30. There were twelve, thirteen, and five persons in our sample population between the ages of 45-60, 61-75, and >75 years (Table 1). The Singh's index is shown in Table 2. Mean vitamin D levels were 9.64+/-3.23 in the femur neck fracture group and 13.42+/-5.31 in the intertrochanteric fracture group, given in Table 3. Mean levels of vitamin D are shown in Figure 1. The comminution of the fracture site in groups of the femur and intertrochanteric fractures was eight and six, respectively.

**Table 1:** Age and fracture type between groups IT: intertrochanteric.

Age in years	Fracture type		Total	p-Value
	Femur neck	IT		
45-60	3	9	12	0.540
61-75	6	7	13	
>75	2	3	5	
Total	11	19	30	

**Table 2:** Singh's index between groups IT: intertrochanteric.

Singh index	Fracture type		Total	Chi-square	p-Value
	Femur neck	IT			
1	3	4	7	5.706	0.127
2	3	2	5		
3	5	6	11		
4	0	7	7		
Total	11	19	30		

**Table 3:** Vitamin D levels between groups IT: intertrochanteric.

Vitamin D levels	Fracture type	N	Mean	Standard deviation	p-Value
	Femur neck	11	9.64	3.233	0.022
	IT	19	13.42	5.316	

**Table 4:** Fracture site comminution and fracture type between groups IT: intertrochanteric.

Fracture site comminution	Fracture type		Total	p-Value
	Femur neck	IT		
No	3	13	16	0.029
Yes	8	6	14	
Total	11	19	30	

## Discussion

Osteoporosis-induced fractures occur in one-third of females and one-quarter of males over 50 years of age. Globally, nearly nine million individuals with osteoporosis have a fracture per year [5]. Muscle fatigue, generalized body pain, decreased strength/balance, increased bone turnover, increased risk of falls, and sustenance of hip fractures are correlated with decreased serum levels of vitamin D in older adults [6]. Increased mortality was observed in patients with hip fractures and high mortality rates were observed in men relative to women. Higher mortality is correlated with institutionalization combined with co-morbidity [7]. The risk of hip and any non-vertebral fractures in outpatient or institutionalized elderly people tends to be minimized by sufficient levels of cholecalciferol or ergocalciferol (700-800 IU/day) [8]. In order to maximize potential benefits and mitigate potential risks, supplementation of vitamin D to restore 25(OH) D levels within a range of 30-50 ng/ml is appropriate. This, of course, should be taken into account in the sense of the patient’s individual needs and co-morbidities [9] and the potential association between decreased mortality and post-fracture use of prescribed supplementation of calcium plus vitamin D and concomitant use of anti-osteoporotic drugs in females. Further investigations are required, however, in order to understand the reasons behind the reduction in the risk of death [10]. The prevalence of hypovitaminosis D in osteoporotic hip fracture patients is high and secondary hyperparathyroidism is observed in more than half of the cases. In patients with low sunlight exposure and low nutritional and functional status, vitamin D deficiency is particularly prevalent [11]. About three-quarters of patients with hip fractures have vitamin D deficiency and two-thirds have secondary hyperparathyroidism. The 25-OH serum level can therefore be a useful index for assessing the risk of hip fracture in India [12]. The 25(OH) D insufficiency over five years was associated with an increased 10-year risk of hip and major osteoporotic fractures in these elderly women [13].

Taken together, our evidence shows that in the etiology of femoral neck fractures, impaired bone mineralization accompanied by low serum 25-(OH) D levels is of significant importance. Consequently balancing serum 25-OH D levels and thus normalizing serum PTH levels may counteract pronounced defects in mineralization and can reduce the incidence of femoral neck fractures [14]. After a fragility fracture, osteoporosis is diagnosed and treated with specific osteoporosis drugs in compliance with the guidelines and, if possible, does not impair the concept of fracture recovery; it substantially decreases the likelihood of future fractures. In order to improve fracture healing, further investigations are needed for approval [15]. In this systematic review and meta-analysis, no decreased risk of fracture was associated with either sporadic or regular dosing with standard doses of vitamin D alone, but a more promising approach was daily supplementation of both vitamin D and calcium [16].

An independent indicator of post-fracture mortality risk in both women and men was a rapid bone loss. The correlation between bone loss and post-fracture mortality was primarily observed in women and men following vertebral fracture and non-hip non-vertebral fracture in women. Whether the bone loss is a marker or plays a role in fracture-related mortality remains to be determined [17]. Non-vertebral fractures have recently been related to the risk of mortality. To better understand which particular fractures and variables contribute to the risk of fractures-associated mortality, larger studies are required. Due to its possible reversibility with anti-fracture therapies, the role of bone in post-fracture mortality needs to be confirmed in more studies [18]. Bisphosphonate and non-bisphosphonate osteoporosis drugs were significantly associated with reduced hip fracture mortality after fragility [19]. Monitoring serum concentrations of 25-hydroxyvitamin D annually can help reveal deficiencies in vitamin D. Responsive exposure to sunlight (usually five-ten minutes of exposure to the arms and legs or hands, arms, and face, two or more three days a week) and increased dietary and supplementary intakes of vitamin D are fairways of ensuring adequate vitamin D [20].

Limitations of the study

The key drawback of our research was the limited sample size and the single center. The present research did not have a control group. In order to further evaluate vitamin D levels and their association with osteoporosis and fracture site comminution in osteoporotic hip fractures, larger randomized controlled trials are needed.

## Conclusions

As part of an overall strategy to improve bone health and to prevent or treat osteoporosis, older women and men should consult their healthcare providers about their nutrient requirements. Recommended levels of vitamin D from foods and supplements should be consumed.

Early diagnosis and treatment of these patients with vitamin D for osteomalacia and anti-osteoporotic osteoporosis regimens will hopefully enhance bone, muscle, and general health, minimizing falls and fractures.
